# Reciprocal Influence of Depressive Symptoms Between Mothers and Fathers During the First Postpartum Year: A Comparison Among Full-Term, Very Low, and Extremely Low Birth Weight Infants

**DOI:** 10.3389/fpsyt.2020.578264

**Published:** 2020-12-08

**Authors:** Erica Neri, Sara Giovagnoli, Federica Genova, Mariagrazia Benassi, Marcello Stella, Francesca Agostini

**Affiliations:** ^1^Department of Psychology, University of Bologna, Bologna, Italy; ^2^Padiatric and Neonatal Intensive Care Unit, Maurizio Bufalini Hospital, Cesena, Italy

**Keywords:** perinatal depression, mothers/fathers, extremely low birth weight, very low birth weight, actor-partner interdependence model (APIM)

## Abstract

**Background:** Perinatal depression (PND) in mothers and fathers of very low and extremely low birth weight (VLBW and ELBW) infants has not been studied extensively. In particular, no studies investigated the reciprocal influence of depressive symptoms during the first 12 months postpartum. This study aimed at exploring the impact of the severity of prematurity on maternal and paternal PND during the first postpartum year; specifically, we used an Actor–Partner Interdependence Model (APIM) to test the interdependence of both partners on depressive symptoms.

**Methods:** A total of 177 mothers and 177 fathers were recruited, divided into 38 couples with ELBW infants, 56 with VLBW, and 83 of full-term (FT) infants. PND was evaluated by the Edinburgh Postnatal Depression Scale (EPDS) at 3, 9, and 12 months postpartum (corrected age for preterm infants).

**Results:** Maternal depressive symptoms at 3 months were positively related to those at 9 and 12 months in the 3 groups. Conversely, paternal depressive symptoms assessed at 3 months were positively related to those measured at 9 months for the ELBW group, 12 months for the VLBW group, 9 and 12 months for FT condition. Furthermore, a significantly positive partner effect was observed regarding the influence of 3 month maternal depressive symptoms on paternal depressive symptoms at 9 months, but only in the case of the VLBW group.

**Conclusion:** Prematurity represents a very specific scenario in the transition to parenthood, leading to specific reactions in mothers and fathers, especially in high-risk conditions. Results should be deepened given the relevance of their clinical implications.

## Introduction

Perinatal depression (PND) is a serious mental disorder, characterized by onset during pregnancy and/or within a year after childbirth (1) and including symptoms such as mood liability, insomnia, disorganized behavior, irritability, and agitation (2). The risk of PND is widely recognized in mothers, with an overall prevalence of about 17% (3–5), but recent literature observed a relevant prevalence also in fathers, with an estimated rate of about 10% (6, 7).

The prevalence of PND could be particularly significant in high-risk contexts, such as in the situation of a preterm birth.

Prematurity, the condition of all births occurring before the 37th week of pregnancy (8), represents an unexpected and stressful event for the parents, who might experience feelings of guilt, grief, and recurrent worries about their baby's survival and health (9–16). The stress experienced can reach such a high intensity that it represents a traumatic experience, in some cases satisfying the criteria to diagnose a post-traumatic stress disorder (17–19).

Both preterm infants' mothers and fathers may also experience high levels of depressive symptoms that could persist (9, 15, 20). Indeed, recent studies found a range of prevalence of PND in preterm babies' mothers of 15–27% in the first 3 months (14, 16, 21, 22), and of 14–21% at 9 and 12 postpartum months (9, 23, 24), confirming that maternal PND after a preterm birth may be significantly more frequent compared to mothers of full-term (FT) infants (20, 22). Recently, an increased interest has been paid also to PND in preterm babies' fathers: nevertheless, to our knowledge, studies are sparse and investigated depressive symptomatology only at 3 months postpartum, reporting 0–6% as a range of prevalence (14, 25).

The risk of PND may be intensified when prematurity is more severe (20, 26). Nevertheless, studies usually focus on low birth weight (LBW) and very low birth weight (VLBW) babies (birth weight <2,500 and 1,500 g, respectively) (14, 27), neglecting the investigation of a more severe preterm birth condition represented by extremely low birth weight (ELBW) (<1,000 g). This population may increase the occurrence for maternal PND, as shown by previous studies (21, 22), where a greater risk for PND emerged in ELBW mothers rather than in VLBW ones. To our knowledge, no studies have explored paternal PND in the case of ELBW infants.

Another relevant issue in the evaluation of PND in parents of preterm infants regards the possible association between maternal and paternal depression. To our knowledge, studies often have focused separately on mothers or fathers, while the reciprocal influence between partners on depressive symptoms has been neglected. Conversely, the association between maternal and paternal PND has been deeply investigated in parents of healthy full-term infants, but giving somewhat inconsistent findings. Indeed, while many researchers found an association between maternal and paternal PND (28–33), others observed a predictive role of only maternal (34–38) or paternal PND on partner's symptomatology (39, 40); again, other studies did not find any significant associations (41–43). One reason for the inconsistency of these results may be represented by the heterogeneity of the methodology. In particular, many different statistical analyses have been used in the studies; quite often, the statistical methods do not seem appropriate for assessing the interdependence and the direction of the relations found between members of dyads (i.e., correlational analysis, MANOVA, or linear regression). In this context, a promising statistical approach could be represented by the Actor-Partner Interdependence Model [APIM; (44, 45)]. Using structural equation modeling, APIM treats data from both dyad members as nested scores within the same group (i.e., the parental couple), providing both the extent to which one partner's independent variable score influences his/her dependent variables score (actor effect) as well as the other partner's dependent variables score (partner effect). Although different methodological and data-analytic approaches are useful in the study of dyads (i.e., multiple regression, multilevel modeling), structural equation modeling is one of the most widely used data-analytic techniques in social and behavioral sciences. To our knowledge, no study assessed the reciprocal influence of perinatal depressive symptoms between mothers and fathers using these statistical models.

Another methodological issue regards the research design. Indeed, many studies on PND usually have a cross-sectional design, assessing mothers and fathers in one step; to our knowledge, only a few studies investigated the evolution or the trajectories of maternal and paternal PND until 6 or 12 months postpartum (30, 37, 38, 40, 42, 46, 47). This lack is particularly evident in literature on preterm parents, where only two studies investigated parental PND longitudinally (15, 25).

Given that the perinatal period ranges from conception to the end of the first postnatal year, reflecting the interval for the arrival of the baby and parental adjustment, the parental affective state should be assessed in a longitudinal perspective.

For the above-mentioned reasons, there is a need of developing more research comparing maternal and paternal PND, assessing both the influence of severity of prematurity and longitudinal effects.

Therefore, this study aimed at investigating the impact of severity of preterm birth on maternal and paternal depressive symptoms at 3, 9, and 12 months of infant's age (corrected for preterm infants). We hypothesized we would find more intense symptoms of PND in the case of a more severe premature birth (ELBW), compared to VLBW and FT conditions, especially in mothers and in the first postpartum period (3 months) postpartum. Also, we aimed at exploring whether the symptoms of PND of each partner at 3 months were associated to the partner's symptoms at 9 and 12 months; specifically, we aimed to measure interdependence within ELBW, VLBW, and FT mothers and fathers applying an APIM model.

We chose to observe parental PND at the specific time points of 3 and 9 months, considered two milestones for infant development and, as a consequence, important moments for parental adjustment; furthermore, we added the assessment of parental PND at 12 months to evaluate parental PND through the entire perinatal period.

## Methods

### Participants and Procedure

The study participants were 354 parents (177 couples). Eighty-three couples were parents of full-term infants, with a birth weight >2,500 g and gestational age >36 weeks (FT group); the remaining 94 were parents of preterm infants. According to infant birth weight, they were differentiated into 56 couples with VLBW infants (weight between 1,000 and 1,500 g) and 38 couples with ELBW infants (weight <1,000 g).

ELBW and VLBW groups were recruited at the Neonatal Intensive Care Unit (NICU) of Bufalini Hospital (Cesena, Italy), while the FT group was recruited at the antenatal classes held at Health Services in the same town. Exclusion criteria were previous or present psychiatric illness, lack of fluency in Italian, and severe neonatal pathologies.

All the assessments took place in Cesena at “Anna Martini” Laboratory (Department of Psychology, University of Bologna) at 3, 9, and 12 months postpartum (T1, T2, and T3, respectively) (corrected age for preterm infants). After providing the written informed consent, all parents fulfilled an *ad hoc* questionnaire (regarding socio-demographic and infant variables) and a self-report questionnaire for the assessment of depressive symptoms. The study was approved by the Ethical Committee of the Department of Psychology (University of Bologna).

### Measures

The Edinburgh Postnatal Depression Scale [EPDS; (48)] is the most widely used instrument for the assessment of perinatal depressive symptomatology. It is a self-report questionnaire, composed of 10 items, exploring the presence of depressive symptoms during the previous 7 days. The EPDS was developed for use by postnatal women (48) and has been implemented in international research for the detection of perinatal depressive symptoms [i.e., (49, 50)]. To date, the EPDS has been translated into more than 60 languages (51). The questionnaire has been subsequently validated for the detection of perinatal depression in men [i.e., (28, 46, 52)].

As recently underlined by the main author (51), the EPDS deliberately does not assess a number of common depressive symptoms that are also common features of typical perinatal adjustment; this increases the possibility of detecting individuals who truly exhibit a depressive state across the perinatal period. The EPDS also includes some indicators of anxiety and omits somatic symptoms of typical depression. For this reason, studies of the factor structure have identified at least two factors, one represented by a “depressive core” and the other focused on “anxiety” (53, 54).

All the items are scored from 0 to 3, providing a total score ranging from 0 to 30, allowing for derivation of both continuous scores (a high score indicates the probable presence of depressive symptoms) and/or dichotomous scores (referring to a cut-off value that enables identification of individuals with depressive symptoms of clinical relevance). In this latter case, for the Italian version of EPDS, Italian validation studies suggested an optimal cut-off of 9/10 for women (55) and 12/13 for men (56). The version for men has been more recognized as a reliable and valid measure for the detection of distress, rather than proper depression, supporting the findings by previous international studies (52, 57). This state of distress would be mainly characterized by unhappiness and anxiety and less by the most common depressive symptomatology.

### Data Analysis

First, according to our first aim, repeated measures ANOVA was run to compare the level of parental PND according to the birth weight (ELBW, VLBW, vs. FT), parental gender (mothers vs. fathers), and time of assessment (3, 9, and 12 months of age). Moreover, the frequencies of depressed parents among ELBW, VLBW, and FT groups at the 3 times of assessment were investigated by Chi-square analysis.

Second, preliminary analyses were carried out to justify the need for further investigation of the relation between maternal and paternal depressive symptomatology via dyadic data analysis. One fundamental principle with dyadic data is that members of a dyad cannot be considered completely independent one from the other because they share and/or develop similarities in some of their psychological attributes (45). Specifically, correlation analyses between the depression levels in mothers and fathers at T1, T2, and T3 were conducted.

To account for the interdependence of dyadic data, we tested two actor-partner interdependence models (APIM). APIM analyses were carried out for exploring, separately for each infant birth weight group, the relation between one parent's levels of depressive symptoms at T1 on his/her own levels of depressive symptoms at T2 and at T3, respectively (that is, actor effect), as well as on the other partner's levels of depressive symptoms at T2 and at T3, respectively (that is, partner effect).

APIMs were estimated using path analysis (maximum likelihood estimation method) that is a special case of structural equation models without latent variables. All the analyses were performed using Lavaan software (58, 59). To test empirically the distinguishability of dyad members by parental gender, an omnibus test of distinguishability has been done for both the T1->T2 model and the T1->T3 model. The coefficients have been tested using Z tests. The APIM test consists of a two-step approach. In the first step, the saturated APIM model looks for significant actor and partner effects. In the second step, the saturated APIM with K parameters (ratio of the partner to actor effect) is computed separately for each parent of the dyad to provide information about the type of dyadic pattern that characterize the effects reported in the model (60). Step 2 was not performed if the absolute standardized values of the actor effects were < 0.10; indeed, weak actor effects combined with strong partner effects would suggest the presence of a partner-only pattern (61). The regular bootstrapping method was used to calculate confidence intervals of k values. Cases with missing data were handled using Full Information Maximum Likelihood estimation (62). Because the standard APIM is a saturated model, it is just-identified and therefore has only one unique solution. A just-identified model has trivially perfect fit; therefore, information about model fit (e.g., RMSEA, CFI, etc.) is uninformative for the standard APIM and is not reported (63). Instead, model evaluation is based on the magnitude and significance of the path estimates.

## Results

### Descriptive Characteristics

Descriptive analyses showed an overall homogeneity among the 3 birth weight groups, except for parity and maternal education variables (Table 1): VLBW mothers were primiparous in a lower percentage, compared to FT and ELBW ones; also, ELBW mothers showed a lower educational level compared to VLBW and FT mothers. To evaluate the effect of parental educational level, marital status, parental age, and parity on EPDS scores, a series of Repeated Measures Analysis of Variance was performed considering time of assessment (3, 9, and 12 months) as a within-subjects factor, birth weight (ELBW, VLBW, and FT groups), parental gender, and specific confounder variables (parental educational level, marital status, parental age and parity) as between-subjects factors. The results showed a non-significant interaction effect for educational level [*F*_(4, 620)_ = 0.89; *p* = 0.47], for parental age [*F*_(6, 626)_ = 1.22; *p* = 0.30], and for parity [*F*_(4, 628)_ = 0.77; *p* = 0.54]. A significant interaction effect was found considering the variable marital status in the model [*F*_(4, 622)_ = 2.55; *p* = 0.04]; to better evaluate the marital status effect on EPDS scores, a separate Repeated Measures ANOVA was performed for each birth weight group. Results showed a significant effect of marital status by parental gender by time of assessment for the FT group [*F*_(2, 153)_ = 3.12; *p* = 0.04], while no significant interaction effect was found for the ELBW group [*F*_2, 63_ = 0.92; *p* = 0.40) or for the VLBW group [*F*_(2, 93)_ = 2.75; *p* = 0.07]. Taking into account these results, we included the marital status as a confounder variable in the APIM models only for FT group, and, following the parsimony principle, the variable was not included in the APIM models for ELBW and VLBW groups.

**Table 1:** Parents and infant characteristics according to categories of birth weight.

	**ELBW (*n* = 38)**	**VLBW (*n* = 56)**	**FT** **(*n* = 83)**	***F*/χ^**2**^**
**Parental characteristics**				
Maternal Age				2.95
Mean (SD) *in years*	34.54 ± 5.15	35.14 ± 5.47	33.07 ± 4.87	
Paternal age				2.00
Mean (SD) *in years*	36.95 ± 5.11	37.49 ± 5.75	35.68 ± 5.25	
Maternal education, *n*(%)				7.33^*^
Primary and secondary	9 (25%)	4 (7.3%)	8 (9.6%)	
school				
High school and	27 (75%)	51 (92.7%)	75 (90.4%)	
university				
Paternal education, *n*(%)				5.93
Primary and secondary	14 (38.9%)	9 (16.4%)	20 (24.4%)	
school				
High school and	22 (61.1%)	46 (83.6%)	62 (75.6%)	
university				
Marital status, *n*(%)				0.69
Married	20 (54.1%)	32 (57.1%)	50 (61.7%)	
Other	17 (45.9%)	24 (42.9%)	31 (38.3%)	
Parity, *n*(%)				17.24^**^
Primiparous	30 (78.9%)	33 (58.9%)	73 (89%)	
Multiparous	8 (21.1%)	23(41.1%)	9 (11%)	
**Infant characteristics**				
Gender, *n*%)				3.34
Male	19 (50%)	36 (64.3%)	41 (49.4%)	
Female	19 (50%)	20 (35.7%)	42 (50.6%)	
Birth weight				1,173.87^**^
Mean (SD) *in grams*	818.89 ± 122.63	1,305.50 ± 145.22	3,489.87 ± 456.66	
Gestational age				987.24^**^
Mean (SD) *in weeks*	27.29 ± 1.99	30.21 ± 2.11	40.04 ± 1.09	
Type of delivery, *n*(%)				40.46^**^
Spontaneous	10 (28.6%)	14 (26.4%)	63 (75.9%)	
Cesarean section	25 (71.4%)	39 (73.6%)	20 (24.1%)	
Twinning, *n*(%)				21.19^**^
Yes	4 (10.5%)	16 (28.6%)	2 (2.4%)	
No	34 (89.5%)	40 (71.4%)	81 (97.6%)	

* *p ≤ 0.05*.

** *p ≤ 0.01*.

Moreover, significant differences among groups emerged for the variables strictly linked to the condition of preterm birth, as expected: birth weight, gestational age, type of delivery, and twinning (Table 1).

### Depressive Symptoms According to Severity of Birth Weight, Time of Assessment, and Parental Gender

Repeated Measures ANOVA showed a significant effect of the interaction between birth weight and time of assessment [*F*_(4, 646)_ = 3.43, *p* = 0.01]: specifically, ELBW parents showed the highest EPDS score at T1 with a considerable decrease of depressive symptoms at T2 and T3. Conversely, despite EPDS mean scores of VLBW and FT groups are lower at 9 and 12 months than those at 3 months, this decrease is slight and less evident than that shown by ELBW (Table 2).

**Table 2:** EPDS mean and categorical scores according to birth weight, time of assessment and parental gender.

	**BW x time of assessment**			**BW x time of assessment x parental gender**						***F***	
				**Mothers**			**Fathers**				
	**T1**	**T2**	**T3**	**T1**	**T2**	**T3**	**T1**	**T2**	**T3**	**BW x time of assessment**	**BW x time of assessment x parental gender**
**EPDS mean scores^a^**										3.43^**^	1.09
ELBW	6.64 ± 0.49	4.84 ± 0.44	3.87 ± 0.41	7.83 ± 0.70	5.71 ± 0.62	4.11 ± 0.58	5.46 ± 0.70	3.97 ± 0.62	3.63 ± 0.58		
VLBW	4.92 ± 0.42	4.10 ± 0.37	4.00 ± 0.35	5.66 ± 0.59	4.44 ± 0.52	4.32 ± 0.49	4.19 ± 0.60	3.75 ± 0.53	3.69 ± 0.50		
FT	4.94 ± 0.33	4.43 ± 0.29	3.92 ± 0.27	5.19 ± 0.45	4.76 ± 0.40	4.19 ± 0.38	4.70 ± 0.47	4.10 ± 0.41	3.65 ± 0.39		
**EPDS mean scores^b^**										***χ*****2**	
ELBW	20 (26.3)	8 (10.5)	5 (6.6)	15 (39.5)	6 (15.8)	5 (14.3)	5 (13.2)	2 (5.3)	0 (0.0)	14.01^**^	/
VLBW	15 (13.4)	6 (5.4)	9 (8.0)	10 (17.9)	6 (11.8)	8 (14.5)	5 (8.9)	0 (0.0)	1 (1.9)	1.38	/
FT	14 (8.4)	12 (7.2)	7 (4.2)	13 (15.7)	8 (9.6)	6 (7.2)	1 (1.2)	4 (4.9)	1 (1.3)	1.98	/

*BW, birth weight; T1, 3 months; T2, 9 months; T3, 12 months*.

a *Values are mean ± SD*.

b *Values are n (%)*.

** *p ≤ 0.01*.

No significant effects were found when we considered the interaction among birth weight, time of assessment, and parental gender [*F*_(4, 646)_ = 1.09, *p* = 0.36].

When we considered the categorical scores of EPDS (depressed vs. non-depressed), a significantly higher frequency of depressed parents emerged at T1 in the ELBW group compared to those of VLBW and FT groups (χ^2^ = 14.01, *p* = 0.01) (Table 2). This result emerged also when analyses were run separately for mothers (χ^2^ = 9.40, *p* = 0.01) and fathers (χ^2^ = 7.43, *p* = 0.02) (Table 2).

No significant differences emerged among the 3 birth weight groups at T2 and T3 (Table 2), neither in the total sample, nor in mothers' and fathers' separate samples.

### Reciprocal Influence of Depressive Symptoms Between Mothers and Fathers

Most of the correlations among maternal and paternal EPDS at T1, T2, and T3 were significant, for each birth weight group (Table 3). Overall, a high correspondence emerged between mothers' and fathers' EPDS scores measured at the same time of assessment. Moreover, mothers' and fathers' depression levels were correlated with their own as well as their partner's depression among the different times of assessment. Results indicated that actor effect, both for mothers and fathers, as well as partner effect could be estimated.

**Table 3:** Pearson correlation analysis on EPDS scores at T1, T2 and T3 in mothers and fathers according to birth weight.

				**Mothers**			**Fathers**	
			**EPDS T1**	**EPDS T2**	**EPDS T3**	**EPDS T1**	**EPDS T2**	**EPDS T3**
**ELWB**	Mothers	EPDS T1	1	0.55^**^	0.38^*^	0.48^**^	0.49^**^	0.21
		EPDS T2		1	0.64^**^	0.38^*^	0.54^**^	0.27
		EPDS T3			1	0.20	0.28	0.55^**^
	Fathers	EPDS T1				1	0.77^**^	0.30
		EPDS T2					1	0.33
		EPDS T3						1
**VLWB**	Mothers	EPDS T1	1	0.67^**^	0.61^**^	0.36^**^	0.33^*^	0.36^**^
		EPDS T2		1	0.84^**^	0.05	0.39^**^	0.39^**^
		EPDS T3			1	0.17	0.44^**^	0.44^**^
	Fathers	EPDS T1				1	0.08	0.57^**^
		EPDS T2					1	0.47^**^
		EPDS T3						1
**FT**	Mothers	EPDS T1	1	0.56^**^	0.46^**^	0.33^**^	0.25^*^	0.12
		EPDS T2		1	0.61^**^	0.18	0.37^**^	0.28^*^
		EPDS T3			1	0.18	0.29^**^	0.31^**^
	Fathers	EPDS T1				1	0.60^**^	0.67^**^
		EPDS T2					1	0.73^**^
		EPDS T3						1

* *p ≤ 0.05*.

** *p ≤ 0.01*.

An omnibus test of distinguishability has been done for both T1->T2 model and T1->T3 model to test empirically the distinguishable factors of dyad members by parental gender. The results of the omnibus test of distinguishability suggest that in our sample the members of the dyad can be considered statistically distinguishable (T1–T2: Chi^2^[6] = 20.54; *p* = 0.01; T1–T3: Chi^2^[6] = 20.54; *p* = 0.01). Therefore, in this study, we conclude that dyad members were distinguishable based on the variable gender.

**Figure 1:**
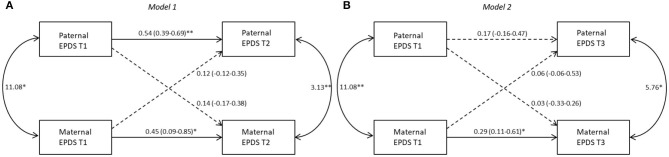
Actor-partner interdependence models for depression in ELBW group 95% CI are reported in parentheses; *p ≤ 0.05; **p ≤ 0.01. *Note*. **(A)** Model 1: T1D->T2D, **(B)** Model 2: T1D->T3D. Black lines represent significant paths, dashed lines represent non-significant paths.

**Figure 2:**
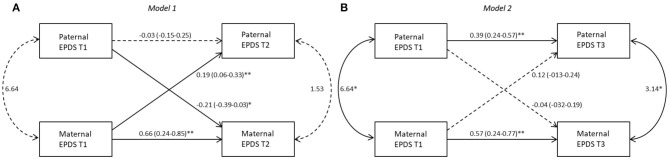
Actor-partner interdependence models for depression in VLBW group 95% CI are reported in parentheses; *p ≤ 0.05; **p ≤ 0.01. *Note*. **(A)** Model 1: T1D->T2D, **(B)** Model 2: T1D->T3D. Black lines represent significant paths, dashed lines represent non-significant paths.

**Figure 3:**
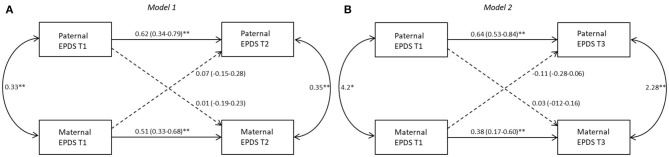
Actor-partner interdependence models for depression in FT group 95% CI are reported in parentheses; *p ≤ 0.05; **p ≤ 0.01. *Note*. **(A)** Model 1: T1D->T2D, **(B)** Model 2: T1D->T3D. Black lines represent significant paths, dashed lines represent non-significant paths.

The results of APIM models based on the different birth weight groups are shown in Figures 1–3. Models 1 and 2 represent, respectively, the evaluation of actor-partner effects estimated on depressive symptoms measured from T1 to T2 (Model 1: T1D->T2D) and those from T1 to T3 (Model 2: T1D->T3D).

#### ELBW Group

##### Model 1

A significant actor effect was found for both mothers (*b* = 0.45, *p* = 0.02) and fathers (*b* = 0.54, *p* = 0.01). No significant partner effect was found from fathers to mothers (*b* = 0.14, *p* = 0.35) nor from mothers to fathers (*b* = 0.12, *p* = 0.39). The k values interpretation suggests that for both mothers (*k* = 0.32, 95% CI [−0.27; 3.86]) and fathers (*k* = 0.22, 95% CI [−0.16; 0.81]) an actor-only model is plausible (Figure 1A).

##### Model 2

A significant actor effect was found for mothers (*b* = 0.29, *p* = 0.02) but not for fathers (*b* = 0.17, *p* = 0.34). No significant partner effect was found from fathers to mothers (*b* = 0.03, *p* = 0.86) nor from mothers to fathers (*b* = 0.06, *p* = 0.68). Interpretation of k values suggests an actor-only model both for mothers (*k* = 0.09, 95% CI [−2.94; 0.88]) and fathers (*k* = 0.38, km = 95% CI [−0.69; 4.38]) (Figure 1B).

#### VLBW Group

##### Model 1

A significant actor effect was found for mothers (*b* = 0.66, *p* = 0.01) but not for fathers (*b* = −0.03, *p* = 0.78). A significant partner effect resulted both for fathers toward mothers (*b* = −0.21, *p*=0.03) as well as for mothers toward fathers (*b*=0.19, *p* = 0.01), meaning that fathers as well as mothers, having a highly depressed partner at T1, reported themselves a higher level of depressive symptoms at T2. However, the k values interpretation suggests an actor-only model (*k* = 0) for mothers: the k value for mothers was equal to −0.31 with a 95% confidence interval ranging from −0.53 to 0.13. Therefore, K parameter for fathers to mothers partner effect was not performed, because the absolute standardized value of the actor effects for fathers was <0.10 (Beta = −0.03), suggesting a partner-only pattern effect (61).

##### Model 2

A significant actor effect emerged for both mothers (*b* = 0.57, *p* = 0.01) and fathers (*b* = 0.39, *p* = 0.01). No significant partner effect was found from fathers to mothers (*b* = −0.04, *p* = 0.73) or from mothers to fathers (*b* = 0.12, *p* = 0.17). The k values interpretation suggests that for both mothers and fathers an actor-only model is plausible (*k* = −0.07, 95% CI [−0.42; 0.8]; *k* = 0.30, 95% CI [−0.27; 0.65], respectively) (Figure 2B).

#### FT Group

Based on the results performed to analyze the impact of possible confounder variables, the marital status has been added at the two subsequent APIM models as between-dyad covariate.

##### Model 1

Results showed a significant actor effect for both mothers (*b* = 0.51, *p* = 0.01) and fathers (*b* = 0.62, *p* = 0.01). No significant partner effect was found from fathers to mothers (*b* = −0.005, *p* = 0.97) or from mothers to fathers (*b* = 0.07, *p* = 0.50). The k values interpretation suggests that for both parents an actor-only model is plausible (*k* = −0.01, 95% CI [−0.36; 0.70]; *k* = 0.11, 95% CI [−0.19; 0.83], respectively). Marital status did not significantly influence EPDS score for mothers (*b* = 0.57, *p* = 0.32) or for fathers (*b* = −0.70, *p* = 0.20) (Figure 3A).

##### Model 2

A significant actor effect has been found for both mothers (*b* = 0.38, *p* = 0.01) and fathers (*b* = 0.64, *p* = 0.01). No significant partner effect was found from fathers to mothers (*b* = 0.03, *p* = 0.74) or from mothers to fathers (*b* = −0.11, *p* = 0.27). Interpretation of k values suggests an actor-only model for both mothers and fathers (*k* = 0.06, 95% CI [−0.31; 0.60]; *k* = −0.17, 95% CI [−0.38; 0.13], respectively). The covariate did not significantly influence the EPDS score for mothers (*b* = 0.42, *p* = 0.41) or for fathers (*b* = −0.55, *p* = 0.30) (Figure 3B).

## Discussion

This study aimed to explore the impact of the severity of prematurity on parental PND during the 1st year after childbirth. One strength of this study was the focus on both mothers and fathers and, specifically, on the reciprocal influence of depressive symptoms between partners in a high-risk context represented by parental adjustment after a preterm birth. To our knowledge, no previous studies have investigated this topic.

First, we compared depressive symptoms in ELBW, VLBW, and FT parents during the 1st year postpartum. Prematurity is widely recognized as a relevant risk factor for parental PND (9, 15, 20), and our study confirmed significantly higher levels of postnatal depressive symptoms in the 1st months, but only for parents of more severe preterm babies (ELBW). In the other preterm group, VLBW, parents showed low and stable levels of depressive symptoms from 3 to 12 months, similar to FT parents. This result stresses the relevance to distinguish among different preterm populations in research and clinical intervention. Indeed, parents' mental state may be especially impaired in case of higher severity of prematurity, and this is supported by our previous studies (21, 22, 64). For ELBW parents, the first postpartum trimester can represent a highly vulnerable period due to baby's health issues and the parental adjustment after discharge from NICU, and these factors do play a key role in increasing the risk for PND (65, 66).

An unexpected result concerns the similarity between mothers and fathers regarding both levels and frequency of PND in all birth weight groups, according to the time of assessment. While previous literature has widely underlined a higher level of depression and a higher prevalence, in the perinatal period, of depressed mothers compared to fathers (31, 39, 67), we did not find significant differences. This result may suggest that the parental adjustment after a preterm birth similarly characterizes both parents, as fathers also may be more actively engaged (68, 69), reducing gender differences.

The second aim of the study was to fit an APIM to investigate, for each birth weight group, whether and how the level of depression at 3 months of each partner was associated to their own level of depressive symptoms (that is, actor effect) and to the partner's levels of depressive symptoms (that is, partner effect) at 9 and at 12 months postpartum.

According to actor effects, we found a significant association between mothers' depressive symptoms at 3 months postpartum and those experienced at both 9 and 12 months: this result emerged for every birth weight group, that is, mothers of preterm and full-term infants, suggesting that the first postpartum months play a crucial role for the depressive risk during the subsequent months. When fathers were considered, actor effects on outcomes at both 9 months and 12 months were observed only for the FT group. Taken together, these results could open up the possibility of identifying sub-groups of mothers and fathers with a higher risk of chronicity since the first postpartum trimester, in line with recent literature (26, 70, 71), enhancing the possibility to promptly implement screening programs as well as therapeutic support for parents. These actions would decrease the risk of negative consequences of chronic depression on an infant's physical and mental health.

Conversely, for preterm fathers, significant actor effects were found only for the association between scores at 3 and 9 months, in the case of ELBW infants, and between 3 and 12 months for the VLBW group. These findings suggest some considerations. In the case of the ELBW group, it may be possible that the severity of the condition makes fathers more vulnerable to depressive symptomatology also at 9 months, especially in the case of PND during the first assessment. Conversely, no significant associations were observed at 12 months, a time point usually characterized by infant's achievement of new important skills (i.e., deambulation and/or first words), allowing him/her to be more autonomous. It may be possible that, due to a change in fathers' representation of their infant, from a “fragile” baby hospitalized in the NICU to a more healthy and competent infant, fathers may feel reassured and more comfortable in their parenting role, with a positive effect on their affective state.

Regarding the case of the VLBW group, the actor effect that we observed in VLBW fathers represents a quite unexpected result. While the association between PND scores at 3 and 12 months would suggest a long-term effect of early symptomatology, the absence of a significant effect at 9 months undermines the plausibility of this explanation. Taken together, these results showed an unclear profile of PND in VLBW fathers, suggesting that also other variables could influence paternal EPDS scores both at 9 and 12 months. Given the lack of studies on PND in preterm infants' fathers, we recommend development of further studies to deeply explore the effect of risk factor in maintaining stable, improving, or worsening PND in these fathers.

When partner effects were considered, a significant association emerged in the VLBW group, where paternal PND at 9 months was significantly influenced by maternal depression at 3 months (partner effect). Some studies have underlined how the prolonged hospitalization of the baby, and quite often of the mother, may weigh on fathers, especially in the case of VLBW, because the active role is expected from them in supporting the partner and taking care of the baby (68, 69). In this context, having a depressed partner could represent an additional factor of pressure for fathers, leading to an increase in distress and depressive symptoms (67).

In all other conditions, no significant partner effect between maternal and paternal PND emerged. The absence of partner effect in the ELBW group may suggest that the higher severity of these infants may represent a traumatic event, where both parents experience more frequently overwhelming negative feelings. For this reason, parents might react by emotionally distancing themselves and becoming less sensitive to the affective states of their partners, with a subsequent absence of significant partner effect.

In our study, a similar reaction could be hypothesized for mothers of VLBW infants. The present results may suggest that VLBW fathers have an active involvement in the care of their infant and their partner, while mothers, as those of ELBW infants, are often more overwhelmed by feelings of sadness, guilt, and failure, as already underlined by literature (11, 72), and may be highly self-absorbed in their suffering and more detached from their partner.

Taken together, these results seem to confirm that ELBW and VLBW parents may differ in the way they cope with the potentially traumatic experience of a preterm childbirth. Furthermore, even if we did not find any influence of parents' gender, these results may suggest that mothers and fathers are characterized by specific reactions and adaptations to their infant's level of prematurity.

Finally, it should be noted that also the results on FT parents showed no reciprocal influence between mothers and fathers. Although we found significant correlations between maternal and paternal EPDS scores, confirming previous literature (28, 29, 31–33), the interdependence between partners did not emerge anymore when we used a more appropriate statistical model (APIM). Furthermore, it is to note that maternal and paternal PND were usually investigated using cross-sectional research studies (29, 31, 32), while in this study the APIM was performed in a longitudinal design.

In summary, the results seem to suggest a specificity of maternal and paternal affective responses to preterm birth, where the influence of a partner's symptomatology on the other's symptoms is only partially present (73–75).

Some limits of the study may be acknowledged. First, the results need to be confirmed on larger samples, also considering a similar size among groups. Second, in the present study we assessed PND through a self-report questionnaire (EPDS): given the limitations of this kind of measure, it may be useful to replicate the study using a clinical interview to diagnose the depressive condition. We may add some more detailed considerations on the use of EPDS. The international literature on the psychometric characteristics of this instrument has underlined how, regarding the fathers' population, the EPDS would show a different factor structure from the EPDS used on mothers. In fact, as reported by previous studies (53, 57), the EPDS for fathers seems more appropriate in detecting a general level of distress given by anxiety, unhappiness, and worry. In the Italian version for fathers by Loscalzo et al. (56), this aspect has been confirmed by a factorial structure characterized by a most prevalent factor, concerning items on unhappiness and anxiety, and only a small portion of the variance explained by a “depressive core.” These characteristics of EPDS could possibly explain why, in our samples, we found very low prevalence rates of clinically depressed fathers. As already put in evidence by Matthey and Agostini (76), all these findings support the evidence that (1) probably this kind of general distress is a more typical expression of emotional maladjustment in men in the first postpartum months, compared to women; (2) considering that the EPDS is the same for both genders, it may be less suited for the identification of perinatal depression in fathers; and (3) there is the need to further analyze the psychometric properties of the EPDS for men.

Taking into account these limitations on the use of EPDS, we underline also the fact that, up to now, the EPDS is the only validated measure available for both mothers and fathers and specifically aimed at detecting the perinatal depressive symptomatology; besides, using the same instrument for both genders, we enable in this study the comparison between the two samples and the comparison with all the massive international literature published on EPDS since 1987.

Another limitation of the study was that we evaluated parental PND longitudinally, but no specific analyses were run to identify the trajectories of symptomatology, as suggested by recent literature (30, 38, 40, 47). Also, in our study we did not investigate anxious symptoms, which are known to occur often in comorbidity with depression and may represent the difficulties in parental adjustment after a preterm birth (67, 77).

Future studies are needed to confirm the results also controlling for the effect of other variables, such as specific characteristics of parental couples (e.g., quality of dyadic relationship, social support), which may interact with parental PND (78, 79). Besides, it would be relevant to study the possible implications of maternal and paternal PND on the quality of caregiving.

Globally, this study suggests that preterm birth represents a very specific scenario in the transition to parenthood, leading to possibly different affective reactions in mothers and fathers for what concerns depressive symptomatology. Given the paucity of the research on the reciprocity between maternal and paternal PND in prematurity, these results may shed new light on this field but would benefit from a confirmation by further studies.

## Data Availability Statement

The raw data supporting the conclusions of this article will be made available by the authors, without undue reservation.

## Ethics Statement

The studies involving human participants were reviewed and approved by Ethical Committee of the Department of Psychology (University of Bologna). The patients/participants provided their written informed consent to participate in this study.

## Author Contributions

EN prepared the study design, organized the sample recruitment, collected data, and contributed to the writing of all sections of the manuscript. SG and FG performed statistical analysis, prepared the tables, contributed to write the manuscript's methods, results, and references sections. MB and MS contributed to prepare the study design and supervised data collection and the research team. FA prepared the study design, supervised all the phases of the research study, and contributed to the writing of all the sections of the manuscript. All authors reviewed and approved the manuscript for publication.

## Conflict of Interest

The authors declare that the research was conducted in the absence of any commercial or financial relationships that could be construed as a potential conflict of interest.
